# Supplementary material for: In Silico Genomic and Metabolic Atlas of Limosilactobacillus reuteri DSM 20016: An Insight into Human Health
Source: Microorganisms. 2022 Jul 2;10(7):1341. doi: 10.3390/microorganisms10071341 (PMC9320016; doi:10.3390/microorganisms10071341)
Supplement: Supplementary file 1 [file microorganisms-10-01341-s001.zip › microorganisms-1755071-supplementary.pdf]

Table S1. Gene table showing all 200 genes identified within the genome. Included are their proposed functions and gene names, organised into their functional pathway groups.

Functions	Sub-group	Gene name	Locus	Function of gene
Propionate metabolism	Propionate metabolism	Glycerol 2-dehydrogenase	Lreu_1840	Converts hydroxy-acetone to 1,2 Propanediol (1,2-PD).
		1,3-propanediol dehydrogenase (EC:1.1.1.202) - iron-containing alcohol dehydrogenase.	Lreu_0030	Conversion of propionaldehyde to propan-1-ol alongside PduQ.
		Acetate kinase	Lreu_0560<br>Lreu_1733	Converts propionyl-P/ propionyl PO4 (2-) to propionate.
		1-propanol dehydrogenase - PduQ	Lreu_1734	Converts propanal/propionaldehyde to propan-1-ol.
		Propionaldehyde dehydrogenase (EC:1.2.1.87)	Lreu_1735	Converts propionaldehyde to propionyl-CoA.
	Cobalamin related	Propanediol utilisation protein - phosphate propanoyl transferase (EC:2.3.1.222) PduL	Lreu_1740	Propionyl-CoA to propionyl-PO4 (2-).
		ATP:cob(I)alamin adenosyltransferase - PduS	Lreu_1736	Involved in cobalamin biosynthesis.
		Cob(I)alamin adenosyltransferase (EC:2.5.1.17) PduO	Lreu_1737	Converts certain substances in coenzyme B12.
	Reuterin/propionate	Hypothetical protein - PduH	Lreu_1743	Gene fused to PduG, small subunit.
		Diol/glycerol dehydratase reactivating factor, large subunit - PduG	Lreu_1744	Codes for glycerol dehydratase activase, replacing inactive cobalamin in glycerol dehydratase.
		Glycerol dehydratase - small subunit PduE	Lreu_1745	Involved in the 1,2-PD to propionaldehyde degradation pathway to propionate. These three subunits form the glycerol dehydratase enzyme.
		Glycerol dehydratase - medium subunit PduD	Lreu_1746	
		Glycerol dehydratase - large subunit PduC	Lreu_1747	
Bacterial Microcompartment protein (BMC)	Pdu operon shell components	Ethanolamine utilisation protein EutP	Lreu_1728	BMC related.
		Ethanolamine utilisation protein EutS	Lreu_1732	Microcompartment related.
		Ethanolamine utilisation protein EutN	Lreu_1738	Microcompartment related.

		Hypothetical protein - propanediol utilisation protein PduM	Lreu_1739	Microcompartment related.
		Microcompartments protein PduJ	Lreu_1741	Similar to PduA, but missing central pore mechanism.
		Microcompartments protein PduK	Lreu_1742	A non-integral microcompartment organisation protein.
		Microcompartment protein PduB	Lreu_1748	Binds the MCP protein shell to the enzymatic core.
		Microcompartment protein PduA/EutM	Lreu_1749	Prevents cell growth arrest, forming the central pore.
		Ethanolamine utilisation protein EutJ	Lreu_1751	BMC related.
Cobalamin synthesis/ B12 coenzyme	Cobalt transport	CbiQ<br>CbiO<br>CbiN<br>CbiM	Lreu_1707<br>Lreu_1706<br>Lreu_1708<br>Lreu_1709	An ABC transporter involved in the transportation of cobalt.
	Synthesis of B12 coenzyme	Glutamyl-tRNA reductase (EC:1.2.1.70)	Lreu_1703<br>HemA	Coenzyme originates from L-glutamate/L-glutamyl-tRNA (glu). Converts glu to glutamate 1-semialdehyde.
		Glutamate-1-semialdehyde 2,1-aminomutase (EC:5.4.3.8)	Lreu_1700<br>HemL	Converts glutamate-1-semialdehyde to 5-aminolevulinate.
		Porphobilinogen synthase (EC:4.2.1.24)	Lreu_1701<br>HemB	Converts 5-aminolevulinate to porphobilinogen.
		Hydroxymethylbilane synthase (EC:2.5.1.61)	Lreu_1702<br>HemC	Converts porphobilinogen to hydroxymethyl-bilane.
		Uroporphyrinogen III methyltransferase / synthase (EC:2.1.1.107; 4.2.1.75) HemD/CobA	Lreu_1712	Converts hydroxymethyl-bilance to uroporphyrinogen III.
		Precorrin-2 dehydrogenase / sirohydrochlorin ferrochelatase (EC:1.3.1.76; 4.99.1.4)	Lreu_1704<br>CysG	Converts uroporphyrinogen III to Precorrin 2. Undergoes anaerobic pathway, converts precorrin 2 to sirohydrochlorin.
		Sirohydrochlorin cobaltochelatase (EC:4.99.1.3)	Lreu_1711<br>CbiK	Cobalt is added within this step, converting sirohydrochlorin to co-sirohydrochlorin.

	Precorrin-2/cobalt-factor-2 C20-methyltransferase (EC:2.1.1.130; 2.1.1.151)	Lreu_1710 CbiL	Converts co-sirohydrochlorin to co-factor 3.
	Precorrin-3B C17-methyltransferase (EC:2.1.1.131)	Lreu_1714 CbiH	Converts co-precorrin 3 to co-precorrin 4.
	Precorrin-4/cobalt-precorrin-4 C11-methyltransferase (EC:2.1.1.133; 2.1.1.271)	Lreu_1716 CbiF	Converts co-precorrin 4 to co-precorrin 5A.
	Cobalt-precorrin 5A hydrolase (EC:3.7.1.12)	Lreu_1715 CbiG	Converts co-precorrin 5A to co-precorrin 5B.
	Cobalt-precorrin-5B (C1)-methyltransferase (EC:2.1.1.195)	Lreu_1719 CbiD	Converts co-precorrin 5B to co-precorrin 6A.
	Precorrin-6A/cobalt-precorrin-6A reductase (EC:1.3.1.54; 1.3.1.106)	Lreu_1713 CbiJ	Converts co-precorrin 6A to co-precorrin 6B.
	Cobalt-precorrin-6B (C15)-methyltransferase (EC:2.1.1.196)	Lreu_1717 CbiT	Converts co-precorrin 6B to co-precorrin 7.
	Cobalt-precorrin-7 (C5)-methyltransferase (EC:2.1.1.289)	Lreu_1718 CbiE	Converts co-precorrin 7 to co-precorrin 8X.
	Precorrin-8X/cobalt-precorrin-8 methyl mutase (EC:5.4.99.61; 5.4.99.60)	Lreu_1720 CbiC	Converts co-precorrin 8X to cobyrinate.
	Cobyrinic acid a,c-diamide synthase (EC:6.3.5.9; 6.3.5.11)	Lreu_1722 CbiA	Converts cobyrinate to cob(II)yrinate a,c diamide.
	Cob(I)alamin adenosyltransferase (EC:2.5.1.17)	Lreu_1725 Lreu_1737 PduO/CobO	Converts cob(II)yrinate to adenosyl cobyrinate a,c diamide. Also adds vitamin B12s to coenzyme.
	Adenosyl Cobyric acid synthase (EC:6.3.5.10)	Lreu_1705 CobQ	Converts adenosyl cobyrinate a,c diamide to adenosyl cobyrinate hexaamide.
	Adenosyl Cobinamide-phosphate synthase (EC:6.3.1.10)	Lreu_1721 CbiB	Converts adenosyl cobyrinate hexaamide to adenosyl cobinamide.
	L-threonine O-3-phosphate decarboxylase (EC:4.1.1.81))	Lreu_1723	Converts L-Threonine phosphate to (R)-1-Amino-propan-2-yl phosphate for use in (EC:6.3.1.10) with Adenosyl cobyrinate hexaamide.
	Adenosylcobinamide kinase / adenosylcobinamide-phosphate guanylyltransferase (EC:2.7.1.156	Lreu_1699 CobU	Converts adenosyl cobinamide to adenosyl cobinamide phosphate then

		2.7.7.62)		to adenosine-GDP-cobinamide.
		Nicotinate-nucleotide--dimethylbenzimidazole phosphoribosyltransferase (EC:2.4.2.21)	Lreu_1695 CobT	Converts dimethyl-benzimidazole to a-Ribazole-5'-P.
		Alpha-ribazole phosphatase (EC:3.1.3.73)	Lreu_1697 CobC	Converts a-ribazole-5'-P to a-ribazole.
		Adenosyl cobinamide-GDP ribazole transferase (EC:2.7.8.26)	Lreu_1698 CobS	Converts a-ribazole and adenosine-GDP-cobinamide to vitamin B12 coenzyme.
Pdu Operon Regulator		Transcriptional regulator, AraC family, two-component system, response regulator	Lreu_1750	Pdu operon encoder, from Lreu_1695 to Lreu_1752, for B12 biosynthesis, propionate and reuterin production.
Sugar uptake	Glucose uptake	Glucose uptake protein	Lreu_0031 Lreu_0418	Glucose intake.
	Glycerol uptake	Glycerol uptake facilitator protein	Lreu_1752	Responsible for glycerol movement.
		Glycerol kinase	Lreu_1065 GlpK	Involved in glycerol uptake and metabolism.
Pyruvate synthesis	D-lactate	D-lactate dehydrogenase (EC:1.1.1.28)	Lreu_0043 Lreu_0683 Lreu_1631 Lreu_1692	Converts D-lactate to pyruvate.
Histamine production	Histamine biosynthesis	Hypothetical protein - HdcB	Lreu_1831	Converts L-histidine to biogenic amine histamine through the hdc gene cluster.
		Histidine carboxylase, pyruvoyl type (EC:4.1.1.22) - HdcA	Lreu_1832	
		Arginine:ornithine antiporter/lysine permease - HdcP	Lreu_1833	
Glycolysis	Glucose -> Pyruvate	Fructokinase/ glucokinase (FK) (EC:2.7.1.2)	Lreu_1206	. Converts glucose to glucose-6-P.
		Glucose-6-phosphate isomerase (PGI) (EC:5.3.1.9)	Lreu_0420	Converts glucose-6-P to fructose-6-P.
		Fructose-bis-phosphate aldolase (FBA) (EC:4.1.2.13)	Lreu_0238	Converts fructose-1,6-BP to glyceraldehyde-3-P and dihydroxyacetone-P.
		Triosephosphate isomerase (tpiA) (EC:5.3.1.1)	Lreu_0171 Lreu_0237	Converts glyceraldehyde-3P to dihydroxyacetone-P

				(glycerone-P) and vice versa.
		Phosphoglycerate mutase (EC:5.4.2.11)	Lreu_0146<br>Lreu_0291	Converts glycerate-3P to glycerate-2P.
		Pyruvate kinase (EC:2.7.1.40)	Lreu_0751<br>Lreu_0457<br>Lreu_1324	Converts phosphoenolpyruvate to pyruvate by transferring a phosphate group.
		Phosphocarrier protein HPr	Lreu_1325	Involved in the phosphotransferase system in forming pyruvate.
	Glucose conversion	Glucose-6-phosphate 1-dehydrogenase	Lreu_1765	Converts a-D-glucose-6P to D-gluco-1,5-lactone-6P.
		6-phosphogluconolactonase (EC:3.1.1.31)	Lreu_0578	Converts D-gluco-1,5-lactone-6P to D-gluconate-6P.
		6-phosphogluconate dehydrogenase (EC:1.1.1.44)(EC:1.1.1.343)	Lreu_1766	Converts D-gluconate-6P to D-ribulose-5P.
		Ribulose-phosphate dehydrogenase (EC:5.1.3.1)	Lreu_1167	Converts D-ribulose-5P to D-xylulose-5P.
		Xylulose-5-phosphate/ fructose-6-phosphate phosphoketolase (EC:4.1.2.9)(EC:4.1.2.22)	Lreu_1686	Converts D-xylulose-5P to D-glyceraldehyde-3P.
		Aldose 1-epimerase (EC:5.1.3.3)	Lreu_1538	Converts α-D-glucose to β-D-glucose and vice versa.
		Phosphoglucomutase (EC:5.4.2.2)	Lreu_0380	α-D-glucose-1P conversion to α-D-glucose-6P and vice versa.
Adhesion/ Colonisation	Sugars	Alpha glucosidase (EC:3.2.1.20)	Lreu_1017	Converts sucrose to D-fructose and D-glucose. Also catalyses the conversion of maltose to D-glucose.
		Sucrose phosphorylase (EC:2.4.1.7)	Lreu_1542	GtfA, an essential protein for sucrose-dependent autoaggregation for biofilm formation.
	Surface proteins	5 peptide-methionine (R) -S-oxide reductase (EC:1.8.4.12) MsrB	Lreu_0188	Msr reverses the loss of biological activity of proteins due to methionine sulfoxide oxidation.

		Peptide-methionine (S)-S-oxide reductase (EC:1.8.4.11) MsrA	Lreu_1198	ABC transporters that are involved in cystine transport.
	Mucus adhesion	L-cystine transport system substrate-binding protein	Lreu_0296	ATP binding protein involved in cystine transport.
		L-cystine transport system ATP-binding protein	Lreu_0295	Putative transmembrane protein.
		L-cystine transport system ATP-binding protein	Lreu_0294	Cystathionine gamma-lyase.
		L-cystine transport system permease protein	Lreu_0293	Elongation factor that influences ribosome binding.
		Ef-Tu	Lreu_0651	Heat-shock protein - chaperonin family.
		GroEL	Lreu_0354	A sortase that modifies surface proteins.
		Sortase A (EC:3.4.22.70)	Lreu_0234	Fibronectin and fibrinogen binding domains.
	Enzyme	Fibronectin-binding A domain protein	Lreu_0932	LPXTG motif and YSIRK motif found on some surface proteins.
	Fibronectin	Fibronectin-binding protein B (LPXTG motif)	Lreu_1258	
		YSIRK peptide	Lreu_1259	
	Cell wall	DltD<br>DltC<br>DltB<br>DltA	Lreu_0249<br>Lreu_0250<br>Lreu_0251<br>Lreu_0252	Dlt operon catalyses the addition of D-alanine residues to lipoteichoic acids in the cell wall. Responsible for cAMP resistance.
Short chain fatty acids	Butanoate metabolism	*FabII-PI cluster*		
		Holo-(acyl-carrier-protein) synthase (AcpS)	Lreu_0254	Converts apo-ACP to holo-ACP (EC:2.8.7.8). Responsible for acyl activation for fatty acid biosynthesis.
		Holo-(carrier-protein)	Lreu_1160<br>Lreu_0990	Utilised by AcpS as a receiver of 4′-PP from coenzyme A.

	Malonyl-CoA-ACP transacylase (FabD)	Lreu_0989	Converts malonyl-CoA + holo-ACP to malonyl-ACP.
	3-oxoacyl-ACP synthase III (FabH)	Lreu_0991	Converts acetyl-CoA + malonyl-ACP to B-ketoacyl-ACP, produces CO2 and H-S-ACP (EC:2.3.1.180).
	3-oxoacyl-ACP synthase II (FabF)	Lreu_0987	Converts malonyl-ACP + acyl-ACP to B-ketoacyl-ACP, produces CO2 and H-S-ACP (EC:2.3.1.179).
	3-oxoacyl-ACP reductase (FabG)	Lreu_0988	Converts B-ketoacyl-ACP + NADPH + H+ to B-hydroxyacyl-ACP + NADH+ (EC:1.1.1.100).
	3-hydroxyacyl-(acyl-carrier-protein) dehydratase (FabZ)	Lreu_0993<br>Lreu_0985	Converts B-hydroxyacyl-ACP to trans 2-enoyl-ACP + H20 (EC:4.2.1.59).
	Enoyl-ACP reductase (FabI)	Lreu_0981	Converts trans 2-enoyl-ACP + NADPH + H+ to Acyl-ACP (1.3.1.9).
	Acyl-ACP thioesterase	Lreu_0335	Converts acyl-ACP to butyric acid and other FAs (EC:3.1.2.14/ 3.1.2.21)
	Transcriptional regulator, MarR family (FabT)	Lreu_0992	Repressor of the FabII operon.
	Acyl-CoA Hydrolase (TesB)	Lreu_0379	A thioesterase converting acyl-CoA + H20 into CoA + carboxylate.
	Butanoate metabolism		
	Thiolase/Acetyl-CoA acetyltransferase (EC:2.3.1.9)	Lreu_0052	Converts 2acetyl-CoA to acetoacetyl-CoA + CoA
	3-hydroxyacyl-CoA dehydrogenase (EC:1.1.1.157)	Lreu_0103	Converts acetoacetyl-CoA to 3-hydroxybutyryl-CoA
	4-hydroxybutyryl-CoA dehydratase/Crotonase (EC:4.2.1.7	Lreu_0887	Converts 3-hydroxybutyryl-CoA to crotonyl-CoA
	Cronoyl-CoA dehydrogenase	-	Converts crotonyl-CoA to butyryl-CoA (EC:1.3.1.86).
Lactate metabolism	L-lactate/Malate dehydrogenase (NAD) (EC:1.1.1.27	Lreu_0194<br>Lreu_0716<br>Lreu_0907<br>Lreu_1272<br>Lreu_1394<br>Lreu_1903	Converts pyruvate to L-lactate.

		Malolactic enzyme - malate dehydrogenase (EC:4.1.1.101)	Lreu_1532	Converts malate to L-lactate.
	Acetate	Acetate kinase (AckA) (EC:2.7.2.1)<br>PduQ	Lreu_0560<br>Lreu_1733	A kinase involved in the phosphate acetyltransferase pathway, that localises with pduQ to convert acetyl-phosphate to acetate and vice versa.
		Phosphate acetyltransferase (EC:2.3.1.8)	Lreu_0398	Converts acetyl-CoA to acetyl-P and vice versa.
		Pyruvate dehydrogenase E1 component alpha subunit (EC:1.2.4.1)	Lreu_0631	Converts pyruvate to 2-hydroxy-ethyl-ThPP and then further converts to S-acetyl dihydrolipoamide-E.
		Pyruvate dehydrogenase E1 component beta subunit (EC:1.2.4.1)	Lreu_0632	Requires coenzyme A and Lipoate for further conversion.
		Pyruvate dehydrogenase E2 (dihydrolipoamide acetyltransferase) (EC:2.3.1.12)	Lreu_0633	Requires FAD and NAD+ to convert S-acetyl dihydrolipoamide-E to acetyl-CoA.
		Dihydrolipoamide dehydrogenase (EC:1.8.1.4)	Lreu_0634	Binds to core acetyltransferase in pyruvate dehydrogenase and begins the process of oxidation of dihydrolipoyl groups.
		Lipoate-protein ligase	Lreu_1677	Converts (R)-Lipoate to (GcvH)-N6-lipoyl-L-lysine and then further to (E2 protein)-N6-lipoyl-L-lysine.
Amino acids and vitamins	Folate	7,8-dihydroneopterin aldolase/epimerase/oxygenase (EC:5.1.99.8)(EC:4.1.2.25)	Lreu_1280	Converts 7,8-Dihydro-monapterin to 7,8-Dihydroneopterin then further to 6-hydroxymethyl-7,8-dihydropterin and glycolaldehyde.
		2-amino-4-hydroxy-6-hydroxymethyldihydropteridine diphosphokinase (EC:2.7.6.3)	Lreu_1279	Converts 6-hydroxymethyl-7,8-dihydropterin to 6-hydroxymethyl-7,8-dihydropterin-P2.
		Dihydropteroate synthase (EC:2.5.1.15)	Lreu_1275	Converts 4-aminobenzoate and 6-hydroxymethyl-7,8-dihydropterin or 6-

			hydroxymethyl-7,8-dihydropterin-P2 to 7,8-dihydrofolate (DHF).
	Dihydrofolate reductase (EC:1.5.1.3)	Lreu_0770	Catalyses numerous reactions between folate and 5,6,7,8-tetrahydrofolate (THF) and 7,8-dihydrofolate (DHF)
	Dihydrofolate synthase/folylpolyglutamate synthase (EC:6.3.2.12)(EC:6.3.2.17)	Lreu_1277	Converts 7,8-dihydropteroate to 7,8-dihydrofolate (DHF) and THF-polyglutamate to THF-L-glutamate then to 5,6,7,8-tetrahydrofolate (THF).
	FolC bifunctional protein	Lreu_0510	Converts 7,8-dihydropteroate to 7,8-dihydrofolate (DHF), then 5,6,7,8-tetrahydrofolate (THF) to THF-L-glutamate and then THF-polyglutamate.
Biotin	Carboxyl transferase subunit a (AccA)<br>Biotin carboxyl carrier protein (AccB)<br>Biotin carboxylase subunit (AccC)<br>Carboxyl transferase subunit B (AccD)	Lreu_0982<br>Lreu_0986<br>Lreu_0984<br>Lreu_0983	Converts acetyl-CoA to malonyl-CoA with ATP (EC:6.4.1.2).
	BioY<br>EcfT<br><br>EcfA1<br>EcfA2	Lreu_0733<br>Lreu_1453<br>Lreu_1663<br>Lreu_1455<br>Lreu_1454	Biotin transport proteins.
	Energy-coupling factor transport system	Lreu_1662<br>Lreu_1661	
	Biotin--acetyl-CoA-carboxylase ligase	Lreu_0734	BirA family transcriptional regulator. Activates biotin and transfers the biotin moiety.
Thiamine	Thiamine-phosphate pyrophosphorylase (EC:2.5.1.3)	Lreu_1015	Converts 4-amino-5-hydroxymethyl-2-methylpyrimidine diphosphate and 2-(2-carboxy-4-methyl-thiazol-5-yl) ethyl phosphate or 5-(2-hydroxyethyl)-4-methylthiazole phosphate to Thiamine phosphate.

	Thiamine phosphate phosphatase (EC:3.1.3.100)	Lreu_1168	Converts thiamine phosphate to either thiamine or thiamine diphosphate/pyrophosphate (TPP).
	Phosphomethylpyrimidine kinase (EC:2.7.1.49 2.7.4.7)	Lreu_1014 Lreu_1271	Converts 4-amino-5-hydroxymethyl-2-methylpyrimidine to 4-amino-5-hydroxymethyl-2-methylpyrimidine diphosphate.
	Hydroxyethylthiazole kinase (EC:2.7.1.50)	Lreu_1013	Converts 5-(2-hydroxyethyl)-4-methylthiazole to 5-(2-hydroxyethyl)-4-methylthiazole phosphate.
Riboflavin	3,4-dihydroxy 2-butanone 4-phosphate synthase (EC:3.5.4.25) (EC:4.1.99.12)	Lreu_0880	Converts GTP to 2,5-Diamino-6-(5-phospho-D-ribosyl amino)-pyrimidin-4(3H)-one. Converts Ribulose 5-phosphate to 3,4-Dihydroxy-2-butanone 4-phosphate.
	Diamino-hydroxyphosphoribosylaminopyrimidine deaminase (EC:3.5.4.26) (EC:1.1.1.193)	Lreu_0878	Converts 2,5-Diamino-6-(5-phospho-D-ribosyl amino)-pyrimidin-4(3H)-one to 5-Amino-6(5-phospho-D-ribityl amino)uracil.
	5-amino-6-(5-phospho-D-ribityl amino) uracil phosphatase (EC:3.1.3.104)	Lreu_0900	Converts 5-Amino-6(5-phospho-D-ribityl amino)uracil to 5-Amino-6-(ribityl-amino)uracil.
	6,7-dimethyl-8-ribityllumazine synthase (EC:2.5.1.78)	Lreu_0881	Converts 5-Amino-6-(ribityl-amino)uracil and 3,4-Dihydroxy-2-butanone 4-phosphate to 6,7-Dimethyl-8-ribityllumazine.
	Riboflavin synthase (EC:2.5.1.9)	Lreu_0879	Converts 6,7-Dimethyl-8-ribityllumazine to Riboflavin.
	Riboflavin kinase (EC:2.7.1.25)(EC:2.7.7.2)	Lreu_0702	Converts Riboflavin to FMN and/or FAD.
Niacin	Transcriptional regulator, DeoR family Nicotinate phosphoribosyltransferase (EC:6.3.4.21)	Lreu_0115	Converts Nicotinate to Nicotinate D-ribonucleotide.

		Lreu_0284	Converts Nicotinate to Nicotinate D-ribonucleoside.
	Purine-nucleoside phosphorylase (EC:2.4.2.1)	Lreu_0114	Converts N-ribosyl-nicotinamide to Nicotinamide.
	5'-nucleotidase (EC:3.1.3.5)	Lreu_1029	Converts Nicotinate D-ribonucleoside to Nicotinate D-ribonucleotide. Converts Nicotinate D-ribonucleotide to N-ribosyl-nicotinamide.
	Nicotinamide-nucleotide amidase (EC:3.5.1.42)	Lreu_0552	Converts Nicotinate D-ribonucleotide to Nicotinate D-ribonucleotide.
	Purine nucleosidase (EC:3.2.2.1)	Lreu_1658	Converts N-Ribosyl-nicotinamide to Nicotinamide.
	Nicotinate-nucleotide adenylyltransferase (EC:2.7.7.18)	Lreu_1235	Converts Nicotinamide D-ribonucleotide to NAD+. Converts Nicotinate D-ribonucleotide to deamino-NAD+.
	NAD+ Kinase (EC:2.7.1.23)	Lreu_0573	Converts NAD+ to NADP+.
	NAD+ synthase (EC:6.3.1.5)	Lreu_0285	Converts Deamino-NAD+ to NAD+.
Pantothenic acid	Type I pantothenate kinase (EC:2.7.1.33)	Lreu_1619	Converts (R)-Pantothenate to (R)-4′-Phospho-pantothenate.
	Phosphopantothenoylcysteine decarboxylase (EC:6.3.2.5)(EC:4.1.1.36)	Lreu_1174	Converts (R)-4′-Phospho-pantothenate to (R)-4′-Phospho-pantothenoyl-L-cysteine then further to 4′-Phospho-pantetheine.
	Pantetheine-phosphate adenylyltransferase (EC:2.7.7.3)	Lreu_0641	Converts 4′-Phospho-pantethine to Dephospho-CoA.
	Dephospho-CoA kinase (EC:2.7.1.24)	Lreu_1245 CoaC	Converts Dephospho-CoA to Coenzyme-A (CoA).
Lysine	Aspartate kinase (EC:1.2.1.11)	Lreu_0611	Converts L-Aspartate to L-4-Aspartyl-phosphate
	Aspartate semialdehyde dehydrogenase (EC:4.3.3.7)	Lreu_0618 Asd	Converts L-4-Aspartyl-phosphate to L-4-Aspartyl

				phosphate.
		4-hydroxy-tetrahydrodipicolinate synthase (EC:4.3.3.7)	Lreu_0615 DapA	Converts L-aspartate 4-semialdehyde to 4-hydroxy-2,3,4,5-tetrahydro-dipicolinate.
		Dihydrodipicolinate reductase (EC:1.17.1.8)	Lreu_0616 DapB	Converts 4-hydroxy-2,3,4,5-tetrahydro-dipicolinate to L-2,3,4,5-tetrahydro-dipicolinate
		2,3,4,5-tetrahydropyridine-2,6-dicarboxylate N-succinyltransferase (EC:2.3.1.117)	Lreu_0613 DapD	Converts L-2,3,4,5-tetrahydro-dipicolinate to N-succinyl-L-2-amino-6-oxopimelate
		Aminotransferase (EC:2.6.1.17)	DapC	Not currently identified. Converts N-succinyl-L-2-amino-6-oxopimelate to N-succinyl-LL-2,6-diaminopimelate
		Succinyl-diaminopimelate desuccinylase (EC:3.5.1.18)	Lreu_0190 Lreu_0377 Lreu_1544 DapE	Converts N-succinyl-LL-2,6-diaminopimelate to LL-2,6-diaminopimelate
		Diaminopimelate epimerase (EC:5.1.1.7)	Lreu_0610 DapF	Converts LL-2,5-Diaminopimelate to meso-2,6-diaminopimelate
		Diaminopimelate decarboxylase (EC:4.1.1.20)	Lreu_0612 LysA	Converts meso-2,6-diaminopimelate to L-lysine
		Aromatic amino acid aminotransferase apoenzyme (EC:2.6.1-)	Lreu_0044 Lreu_0617 Lreu_0744 Lreu_1200	Converts N-acetyl-L-2-amino-6-oxopimelate to N-acetyl-LL-2,6-diaminopimelate.
		N-acetyl diaminopimelate deacetylase	Lreu_0614	Converts N-acetyl-LL-2,6-diaminopimelate to LL-2,6-diaminopimelate.
Secretion pathway	Sec pathway	Protein translocase subunit SecE/Sec61 gamma	Lreu_0306	Sec-SRP: general secretory protein pathway. IMP.
		Preprotein translocase subunit SecG	Lreu_0392	
		Preprotein translocase subunit SecY	Lreu_1463	
		Preprotein translocase subunit YajC	Lreu_0530	
		YidC/Oxa1 family membrane protein insertase	Lreu_1223 Lreu_1942	

		Protein translocase subunit SecA	Lreu_0365	ATPase.
		Fused signal recognition peptide FtsY	Lreu_1157	SRP receptor.
		Signal recognition particle subunit SRP54 (EC:3.6.5.4) - Ffh	Lreu_1155	Targeting protein.
		YlxM/p13 family protein	Lreu_1156	Potentially regulatory.
GABA shunt	TCA	Fumarate reductase flavoprotein subunit	Lreu_1530	Converts succinate to fumarate.
		Fumarate hydratase	Lreu_1531	Converts fumarate to malate.
	GABA	Succinate-semialdehyde dehydrogenase	Lreu_0034	Converts L-glutamate to 4-aminobutanoate (GABA).
		4-aminobutyrate aminotransferase	Lreu_0199	Converts 4-aminobutanoate (GABA) to succinate aldehyde.

Table S2. Results from a phage display screening showing extracellular and transmembrane proteins. Adapted from Wall et al., 2003 [59] to include the likely identification of these genes (Likely Name/Function) on the current genome assembly using Blast Sequence Similarity Search. The E-value score is the number of hits similar to the query that could be due to chance. The bit-score is the result of a formula that aligns the possible residue pairs and reports a score.

Gene Name	Actual Identification	Likely Name/Function	Accension Number and Proposed Name/Function (Wall et al., 2003)	E-value and bits
Transport				
01	Lreu_0098	Amino acid ABC transporter membrane protein	AY268984 - Putative glutamine ABC transporter	281 bits, 100% identity, 2e-94 e-val
02	Lreu_1419	Hypothetical protein	AY268987 - Putative ABC transporter	125 bits, 100% identity, 8e-38 e-val
03	Lreu_1568	Phosphate ABC transporter substrate-binding protein, PhoT family	AY268991 - Putative phosphate ABC transporter	390 bits, 99% identity (query - L, subject, Q (aa), 5e-139 e-val
04	Lreu_1691	ABC transporter related	AY268996 - Putative ABC transporter	87 bits, 100% similarity, 4e-23 eval
05	-	-	No close similarities. Putative iron chelatin ABC transporter	Closest was 1840 - glycerol 2-dehydrogenase at only 31% identity.
06	Lreu_1508	ABC transporter	Putative ABC transporter ATP-binding protein CydC	115 bits, 100% identity, 4e-33 e-val
Enzyme				
07	Lreu_1842	D-Ala-D-Ala carboxypeptidase A	Putative D-alanyl-D-alanine carboxypeptidase	317 bits, 100% identity, 2e-109, e-val
08	Lreu_1403	N-acetylmuramoyl-L-alanine amidase	Putative autolysin	316 bits, 100% identity, 3e-100 e-val
09	Lreu_1841	Protein of unknown function DUF915, hydrolase family protein	Putative cell surface hydrolase	476 bits, 100% identity, 3e-172 e-val
10	Lreu_1812	Mannosyl-glycoprotein endo-beta-N-acetylglucosaminidase	Autolysin like protein	409 bits, 100% identity, 1e-147 e-val
11	Lreu_0642	Secreted protein containing a PDZ domain-like	Putative endopeptidase	330 bits, 100% identity, 3e-115 e-val
12	Lreu_1314	Peptidoglycan binding LysM	Putative endolysin	339 bits, 100% identity, 1e-119 e-val

13	Lreu_0937	Protein of unknown function DUF915 - hydrolase/endolysin	Putative cell surface hydrolase	343 bits,100% identity, 6e-121 e-val
14	Lreu_1924	Beta-lactamase	Putative Penicillin-binding protein class C fmt-like protein	351 bits, 97% identity (differences in a few aa), 3e-123 e-val
15	Lreu_1853	Mannosyl-glycoprotein endo-beta-N-acetylglucosaminidase	Putative muramidase	656 bits, 100% identity, 0.0 e-val
16	Lreu_1312	Peptidoglycan binding LysM	Cell-wall hydrolase LytN-like protein	34 bits, 100% identity, 4e-118 e-val
17	Lreu_1265	PpiC-type peptidyl-prolyl cis-trans isomerase	Putative peptidylprolyl isomerase	261 bits, 100% identity, 3e-89 e-val
Host/ Microbial Interaction Proteins				
18	Lreu_0415	Peptidoglycan-binding LysM	Apf1-like protein	394 bits, 100% identity, 7e-142 e-val
Cnb	Lreu_0296	ABC transporter substrate-binding protein (Likely MapA)	L. reuteri Cnb gene	530 bits, 100% identity, 0.0 e-val
19	Lreu_0859	N-acetylmuramoyl-L-alanine amidase	PspC-like protein	416 bites, 99% identity, 4e-157 e-val
Sensor- regulator proteins				
20	Lreu_0020	Pas/Pac sensor signal transduction histidine kinase	Putative histidine protein kinase sensor protein	209 bits, 100% identity, 3e-66 e-val
21	Lreu_1259 or Lreu_0095	YSIRK Gram-positive signal peptide or pseudogene	putative extracellular protein	68.2 bits, 45% identity and 4e-15 e-val.<br><br>0095 - listed as pseudogene and no match in blast even with its own sequence.
22	Lreu_1816	NLP/P60 Protein	Putative LysM domain containing protein	198 bits, 98% identity, 9e-64 e-val
23	Lreu_1844	ErfK/YbiS/YcfS/YnhG family protein	Hypothetical cell surface protein	251 bits, 100% identity, 1e-86 e-val
24	Lreu_1901	Protein of unknown function DUF1002/ Extracellular protein	Extracellular protein	505 bits, 100% identity, 0.0 e-val
26	Lreu_0021	YycH protein	putative extracellular protein	374 bits, 98% identity, 1e-130 e-val

Unconserved hypothetical proteins

27	Lreu_1088	Hypothetical protein	Hypothetical extracellular protein	104 bits, 100% identity, 4e-31 e-val
28	Lreu_0028	Hypothetical protein	Hypothetical extracellular protein	152 bits, 100% identity, 1e-50 e-val
29	Lreu_1364	NLP/P60 Protein	Hypothetical extracellular protein	241 bits, 100% identity, 1e-79 e-val
30	Lreu_1642	Hypothetical protein	hypothetical extracellular protein	88.2 bits, 93% identity, 2e-23 e-val
31	Lreu_1587	Peptidase m10A and M12B, matrixin and adamlysin	Hypothetical extracellular protein	313 bits, 99% identity, 2e-110
32	Lreu_0046	Hypothetical protein	Hypothetical extracellular protein	673 bits, 100% identity, 0.0 e-val
33	Lreu_0959	LPXTG-Motif cell wall anchor domain	Hypothetical extracellular protein	481 bits, 99% identity, 4e-174 e-val
34	Lreu_1357	Pseudogene	Hypothetical extracellular protein	100% identity
35	Lreu_1141	Hypothetical protein	Hypothetical extracellular protein	276 bits, 98% identity, 5e-97 e-val
36	Lreu_1094	N-acetylmuramoyl-L-alanine amidase, family 2	Hypothetical extracellular protein	174 bits, 100% identity, 7e-55 e-val
Transport proteins				
37	Lreu_1558	H+ - transporting two sector ATPase	putative sodium ATP synthase subunit	360 bits, 99% identity and 2e-125
38	Lreu_1067	cation:H+ antiporter 2, CPA2 family	putative Na(+)/H(+) antiporter (	221 vits, 100% identity, 7e-71
39	Lreu_0180	heavy metal translocating P-type ATPase	putative cadmium-/zinc-/cobalt-transporting ATPase	570 bits, 99% identity, 0.0 e-val
40	Lreu_1764	Major facilitator superfamily MFS_1	putative transport protein	172 bits, 100% identity, 3e-54 e-val
41	Lreu_1681	branched-chain amino acid transport system II carrier protein	putative branched-chain amino acid transport protein	326 bits, 100% identity, 9e-112 e-val
42	Lreu_1599	Putative hydroxymethylpyrimidine transporter CytX	putative permease	320 bits, 100% identity, 9e-111 e-val
43	Lreu_0161	Major facilitator superfamily MFS_1	putative transmembrane transport protein	315 bits, 97% identity, 6e-108

44	Lreu_1672	Major facilitator superfamily MFS_1	hypothetical transport protein	194 bits, 100% identity, 8e-63 e-val
45	Lreu_0385	Serine/ threonine exchange transporter	putative amino acid transport protein	526 bits, 99% identity, 0.0 e-val
46	Lreu_1861	Mn2+/Fe2+ transporter, NRAMp family	putative manganese transport protein	211bits, 100% identity, 8e-68 e-val
47	Lreu_1861	Mn2+/Fe2+ transporter, NRAMp family	putative manganese transport protein	262 bits, 100% identity, 7e-87 e-val
Enzymes				
48	Lreu_1373	Hypothetical protein	Eps4N-like protein	698 bits, 99% identity, 0.0 e-val
49	Lreu_0588	Phospho-N-acetylmyramoyl-pentapeptide transferase	putative phospho-N-acetylmuramoyl-pentapeptide-transferase	144 bits, 97% identity, 2e-44 e-val
50	Lreu_0521	CDP-diacylglycerol-glycerol-3-phosphate 3-phosphatidyltransferase	putative CDP-diacylglycerol-glycerol-3-phosphate 3-phosphatidyltransferase	278 bites, 100% identity, 3e-97 e-val
51	Lreu_1709	Cobalamin biosynthesis protein CbiM	putative cobalamin biosynthesis protein	156 bits, 100% identity, 6e-50 e-val
Conserved hypothetical proteins				
52	Lreu_0905	LrgA family protein	putative membrane protein	159 bits, 100% identity, 3e-52 e-val
Unconserved hypothetical proteins				
53	Lreu_0170	Hypothetical protein	Hypothetical membrane protein	180 bits, 100% identity, 2e-60 e-val
